# Supplementary material for: Ternary Cu(II) Complex with GHK Peptide and Cis-Urocanic Acid as a Potential Physiologically Functional Copper Chelate
Source: Int J Mol Sci. 2020 Aug 27;21(17):6190. doi: 10.3390/ijms21176190 (PMC7503498; doi:10.3390/ijms21176190)
Supplement: Supplementary file 1 [file ijms-21-06190-s001.pdf]

Supplementary Materials for:

Ternary Cu(II) complex with GHK peptide and *cis*-urocanic acid as a potential physiologically functional copper chelate

Karolina Bossak-Ahmad [1], Marta D. Wiśniewska [1], Wojciech Bal [1], Simon C. Drew [1,2] and Tomasz Frączyk [1,*]

[1] Institute of Biochemistry and Biophysics, Polish Academy of Sciences, Pawińskiego 5a, 02-106 Warsaw, Poland
[2] Department of Medicine (Royal Melbourne Hospital), The University of Melbourne, Melbourne, Victoria 3010, Australia
* Correspondence: tfraczyk@ibb.waw.pl

1. FIGURES

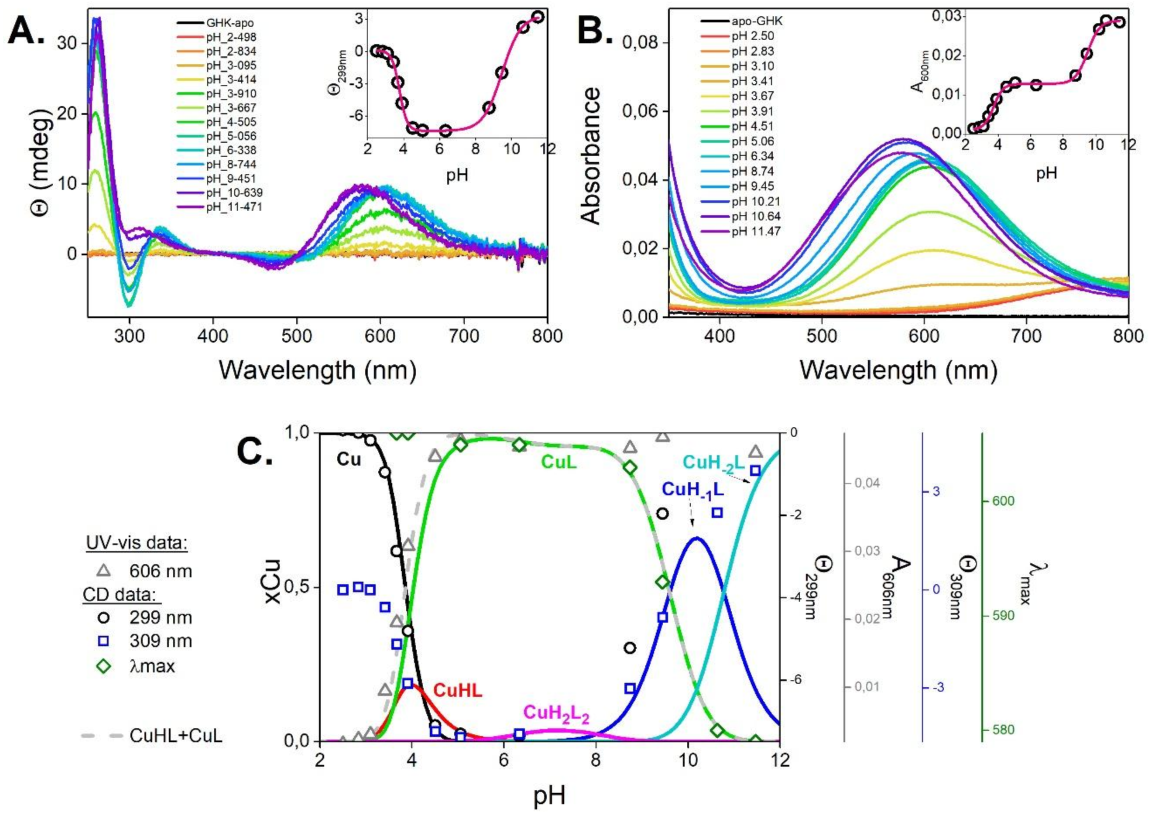

Figure S1. (A) CD and (B) UV-vis spectra of 0.95 mM GHK with 0.8 mM $CuCl_2$ titrated in a pH-metric manner at 25 °C. Spectra are color coded from red (low pH) to blue (high pH). The black line corresponds to the apo-peptide. Insets: the pH-dependent ellipticity at 299 nm and absorbance at 500 nm were fitted with the Hill equation (*pink line*). (C**)** Species distribution diagram calculated using the protonation and stability constants obtained from potentiometric titrations performed under the same conditions. Left abscissa: fraction of Cu(II) complex. Right abscissa: ellipticity and absorbance at discrete wavelengths, and λ_{max} (derived from CD spectra). The dashed-gray line is a summation of the 3N complexes CuHL and CuL.

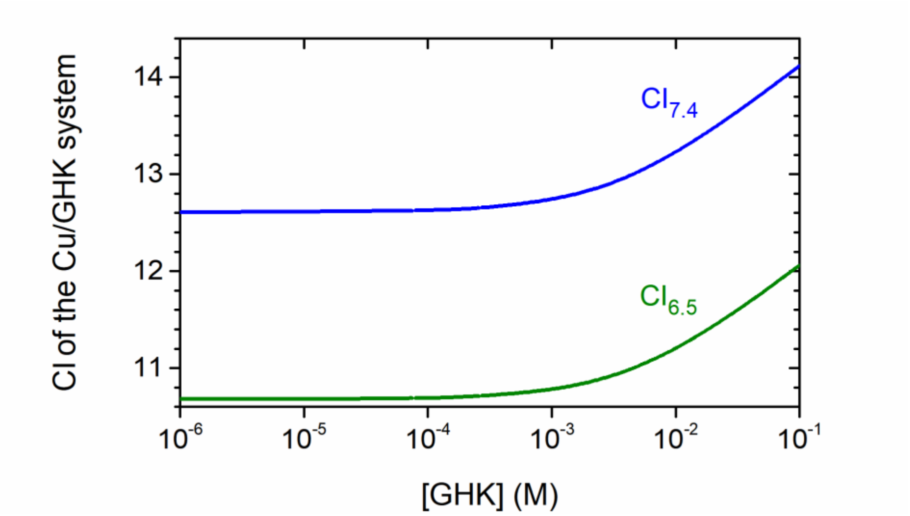

Figure S2. The influence of variable concentrations of GHK on the apparent affinity of GHK to Cu(II). Values of $CI_{7.4}$ and $CI_{6.5}$ are valid for [Cu] = 1 μM, at pH 7.4 and 6.5, respectively.

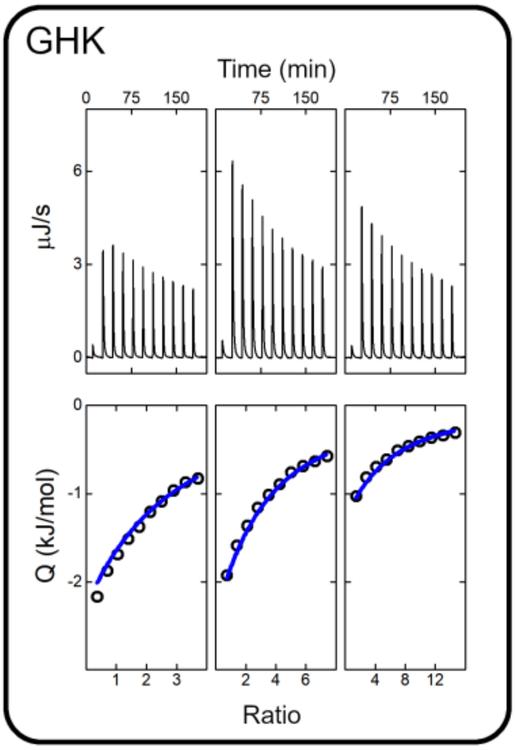

Figure S3. ITC titrations of GHK into solutions containing Cu(GHK). Buffered solutions (20 mM HEPES, 100 mM NaCl, pH 7.4) of 1:0.97 of GHK:$CuCl_2$ was titrated with GHK in a range of 0.4 to 15 mol equiv., at 25 °C. The volume of each injection was 24 μL, with concentration of GHK in syringe 6 to 12 mM GHK. The initial concentration of GHK in cell was 0.5, 0.5, and 0.25 mM, respectively, from left. The upper plots show the raw experimental data. The lower plots show heat in each injection (open dots) with fits (blue lines) of ${}^{C}K_{7.4}$ for the ternary Cu(GHK)(GHK) complex.

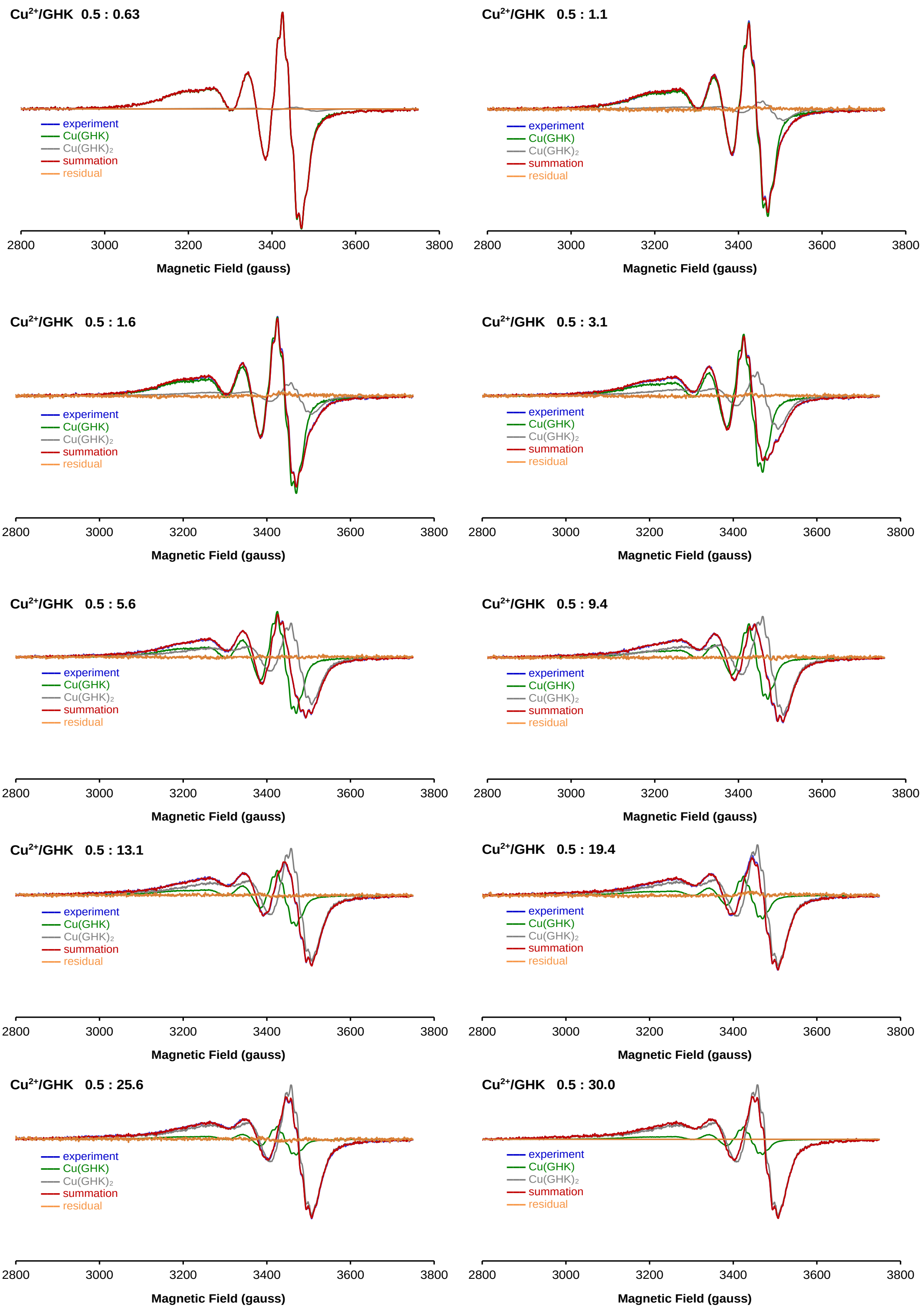

Figure S4. Decomposition of the X-band EPR spectra of Cu/GHK 0.5:*n* (*n* = 0–30 mM). The corresponding percentages of each species are shown in Figure 1 (main text). *Blue spectrum*, experiment; *green spectrum*, Cu(GHK); *grey spectrum*, Cu(GHK)$_2$; *red spectrum*, summation of Cu(GHK) and Cu(GHK)$_2$ spectra; *orange spectrum*, difference between the experimental spectrum (blue) and the summation (red).

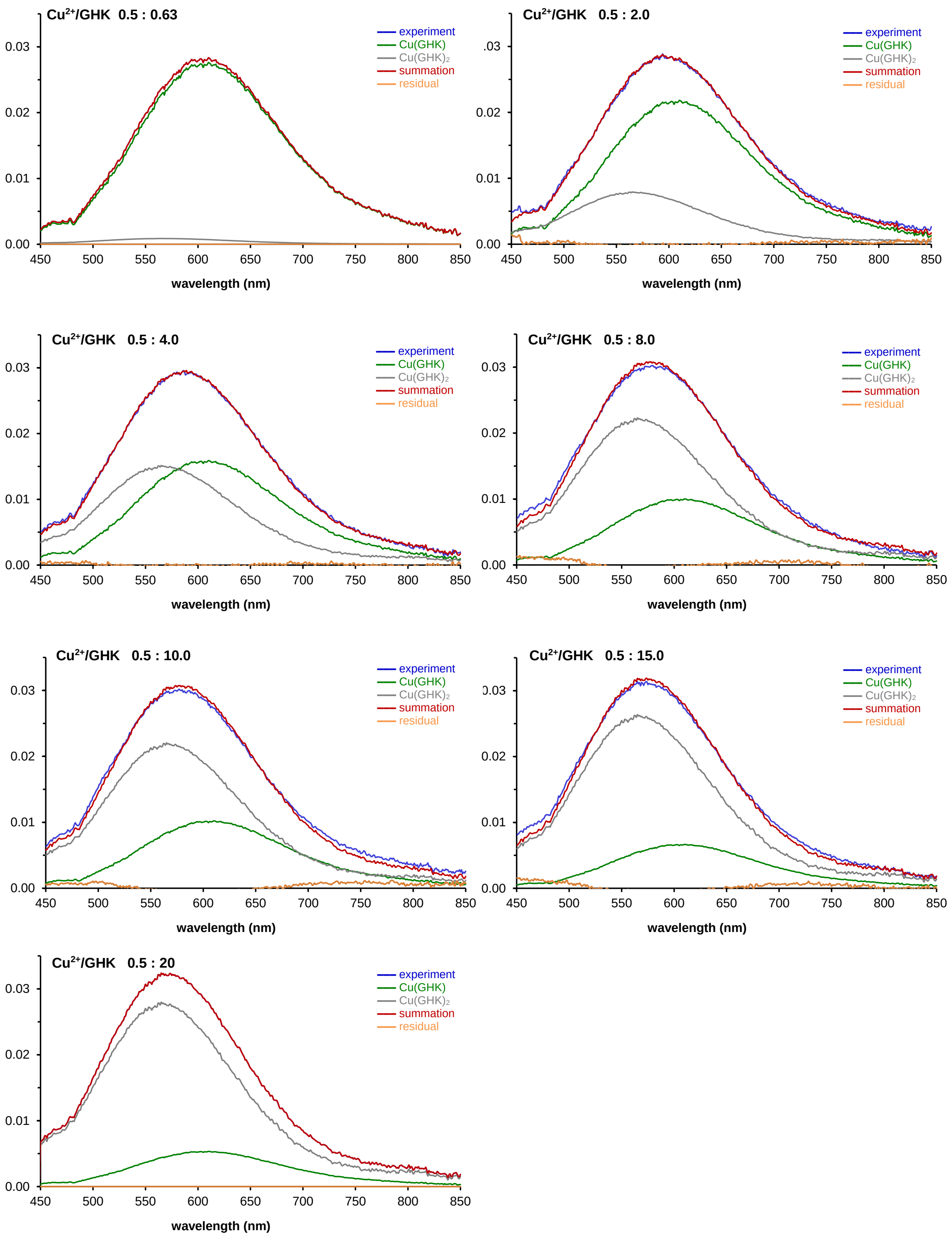

Figure S5. Decomposition of the UV-vis spectra of Cu/GHK 0.5:*n* (*n* = 0–20). The corresponding percentages of each species are shown in Figure 1 (main text). *Blue spectrum*, experiment; *green spectrum*, Cu(GHK); *grey spectrum*, Cu(GHK)$_2$; *red spectrum*, summation of Cu(GHK) and Cu(GHK)$_2$ spectra; *orange spectrum*, difference between the experimental spectrum (blue) and the summation (red).

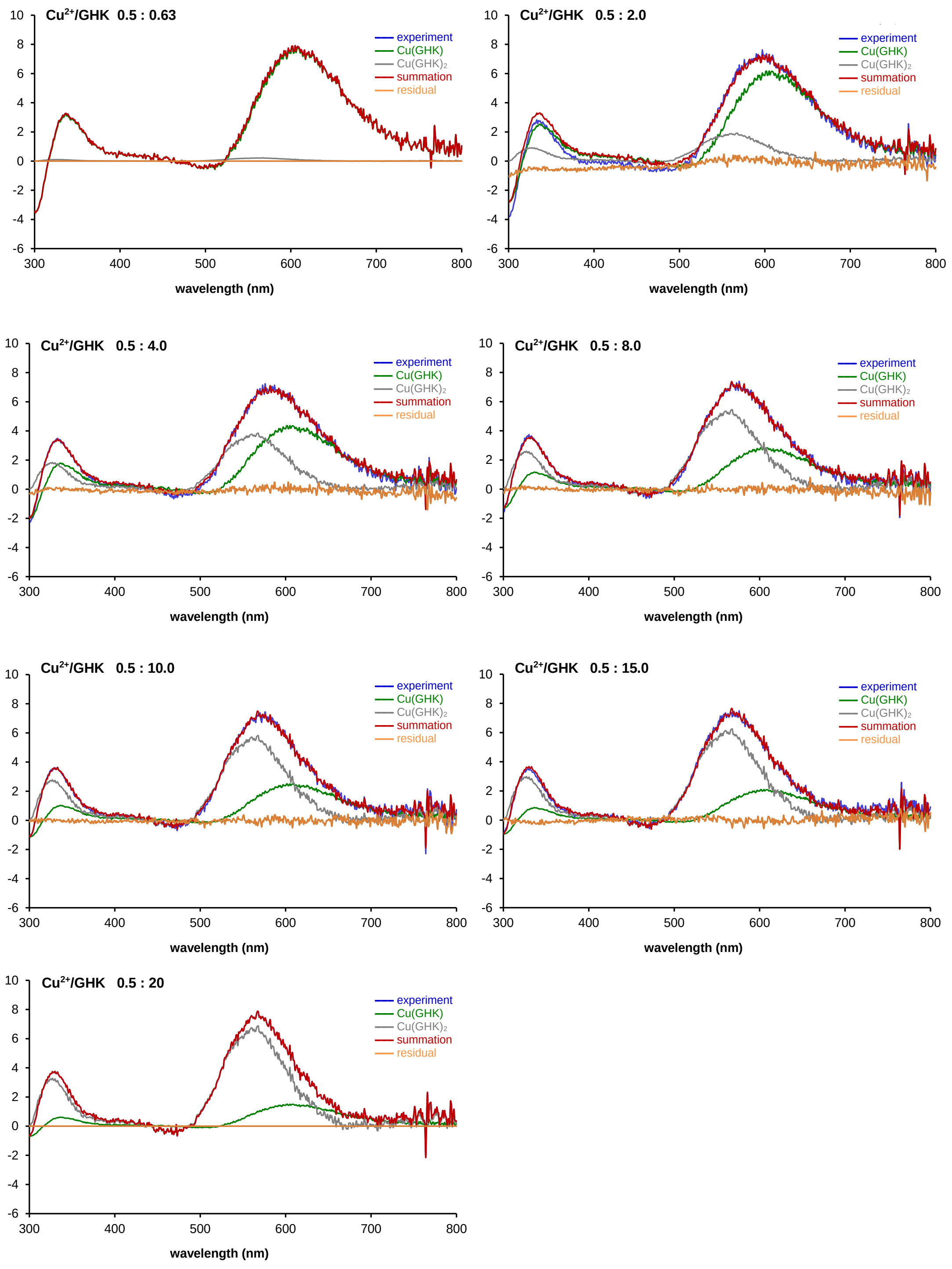

Figure S6. Decomposition of the CD spectra of Cu/GHK 0.8:n (n = 0–20). The corresponding percentages of each species are shown in Figure 1 (main text). *Blue spectrum*, experiment; *green spectrum*, Cu(GHK); *grey spectrum*, $Cu(GHK)_2$; *red spectrum*, summation of Cu(GHK) and $Cu(GHK)_2$ spectra; *orange spectrum*, difference between the experimental spectrum (blue) and the summation (red).

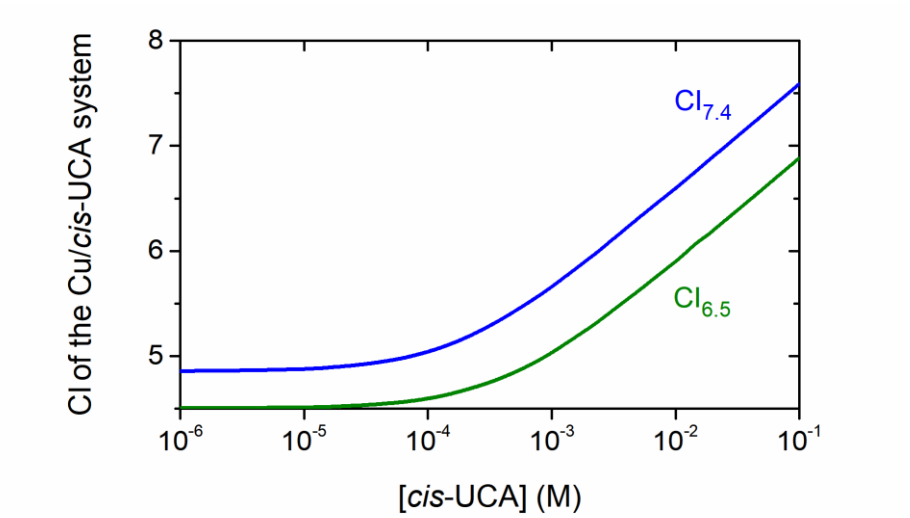

Figure S7. The influence of variable concentrations of *cis*-UCA on the apparent affinity of *cis*-UCA to Cu(II). Values of $CI_{7.4}$ and $CI_{6.5}$ are valid for [Cu] = 1 μM, at pH 7.4 and 6.5, respectively.

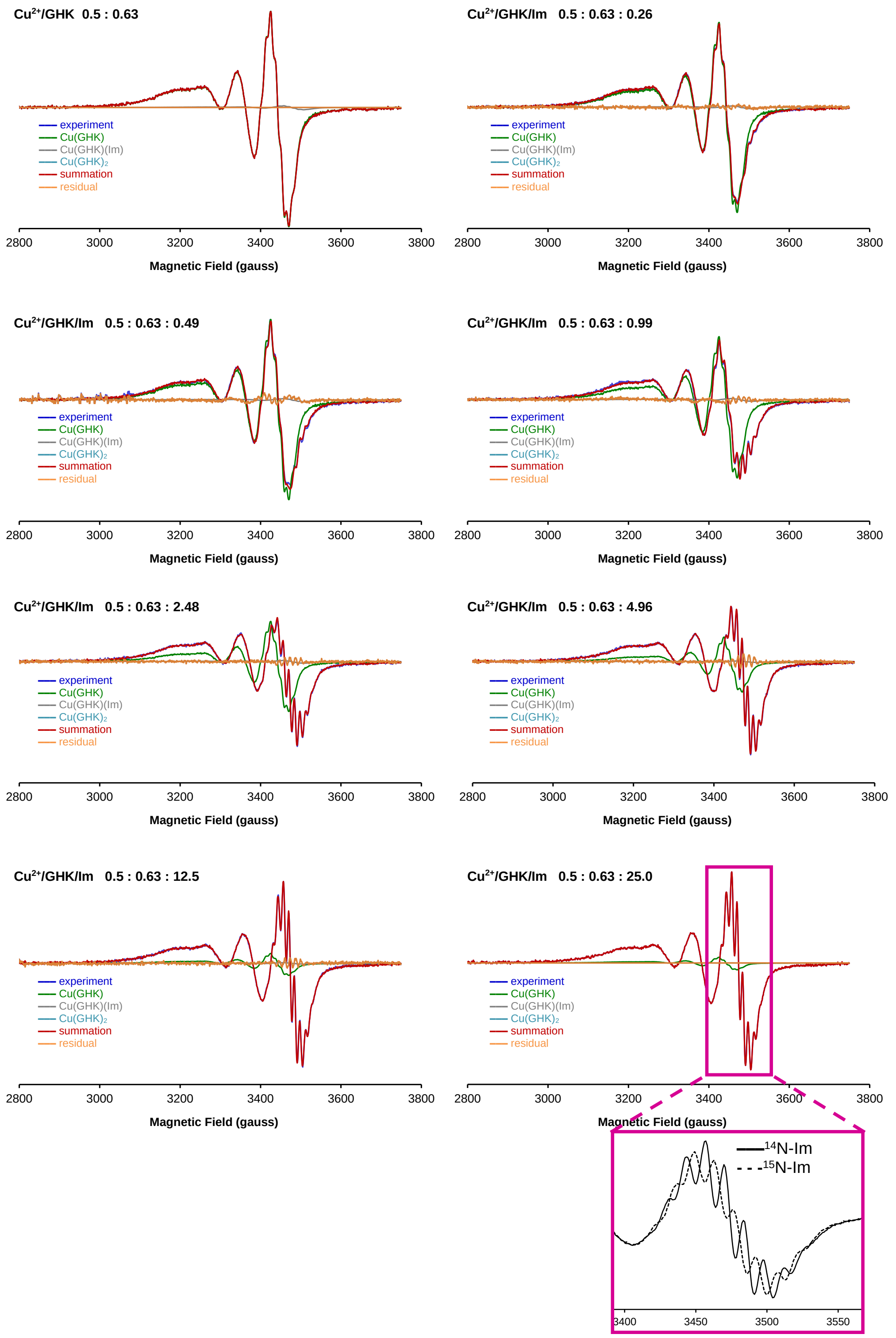

Figure S8. Decomposition of the X-band EPR spectra of Cu/GHK/Im 0.5:0.63:*n* (*n* = 0–25 mM). The corresponding percentages of each species are shown in Figure 3 (main text). *Blue spectrum*, experiment; *green spectrum*, Cu(GHK); *grey spectrum*, Cu(GHK)(Im); *cyan spectrum*, Cu(GHK)$_2$; *red spectrum*, summation of Cu(GHK), Cu(GHK)(Im) and Cu(GHK)$_2$ spectra; *orange spectrum*, difference between the experimental spectrum (blue) and the summation (red). Inset: replacing ^{14}N-imidazole with ^{15}N-imidazole changes the ligand hyperfine pattern for Cu/GHK/Im 0.5:0.63:25 as expected for an equatorial Im ligand in the tetragonal Cu(GHK)(Im) species.

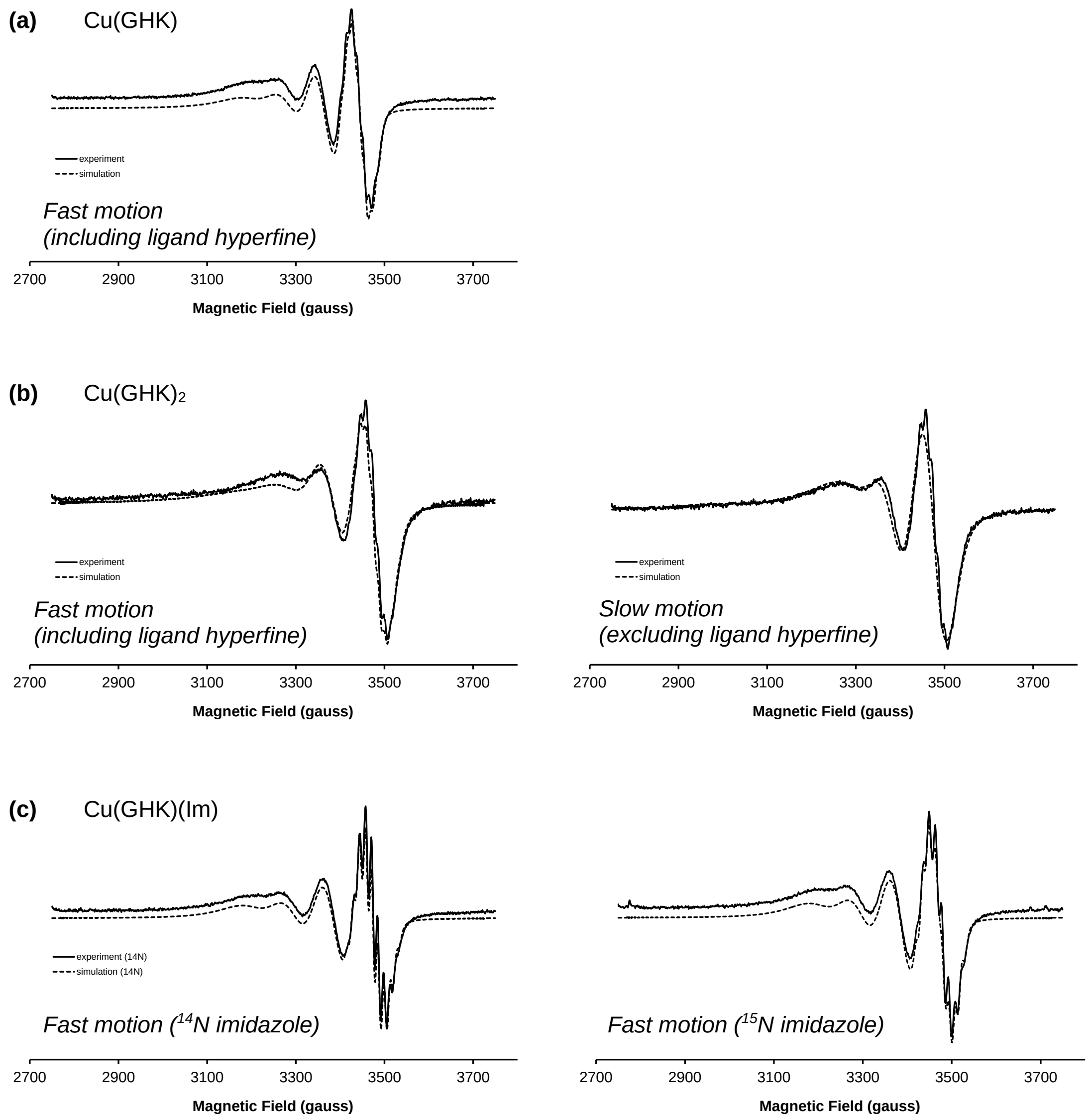

Figure S9. Experimental (———) and simulated (- - - -) room temperature EPR spectra using the spin Hamiltonian parameters listed in Table 1.

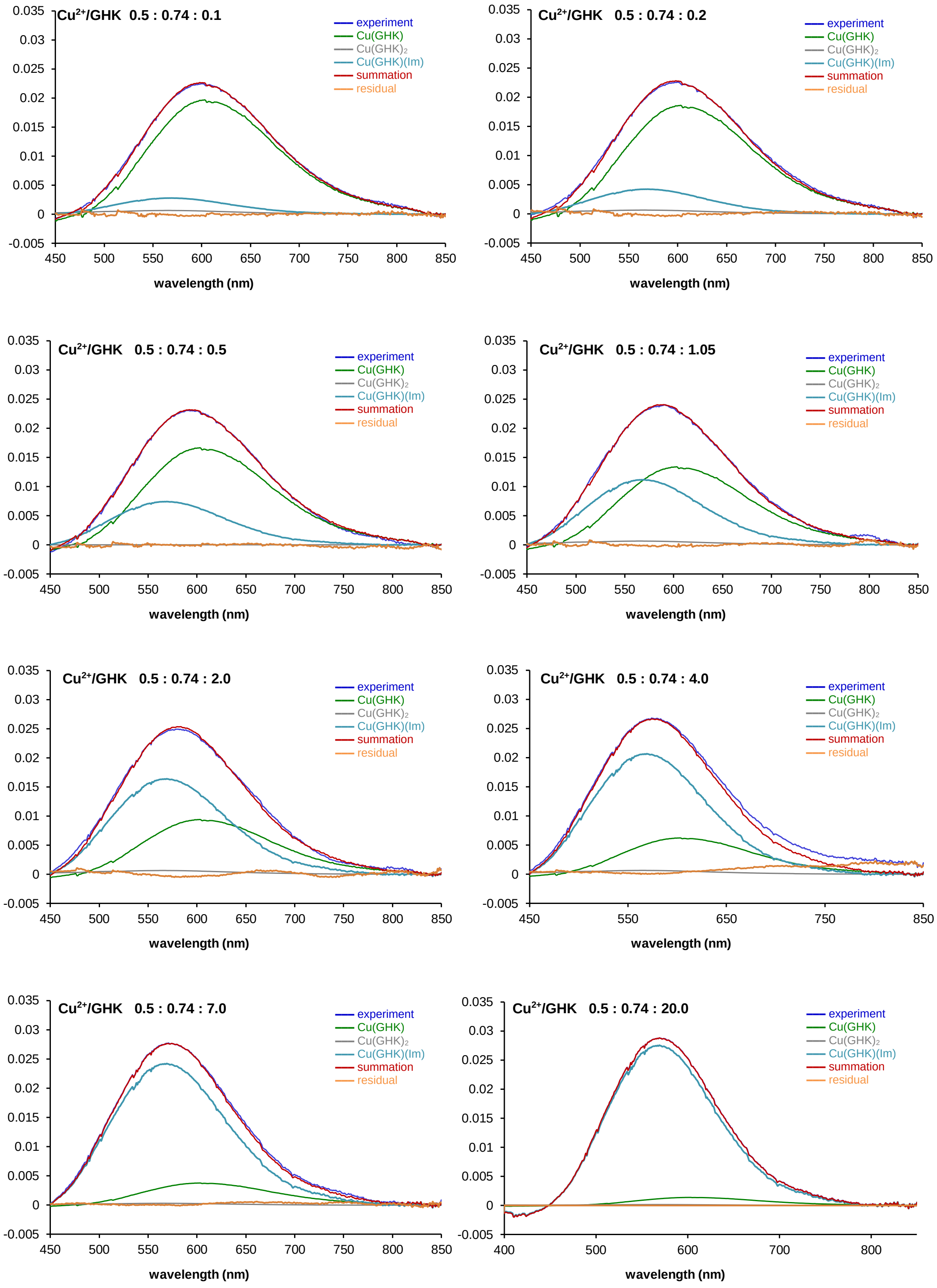

Figure S10. Decomposition of selected UV-vis spectra of Cu/GHK/Im 0.5:0.74:*n* (*n* = 0–20 mM). The corresponding percentages of each species are shown in Figure 3 (main text). *Blue spectrum*, experiment; *green spectrum*, Cu(GHK); *grey spectrum*, $Cu(GHK)_2$; *cyan spectrum*, Cu(GHK)(Im); *red spectrum*, summation of Cu(GHK), $Cu(GHK)_2$, and Cu(GHK)(Im) spectra; *orange spectrum*, difference between the experimental spectrum (blue) and the summation (red).

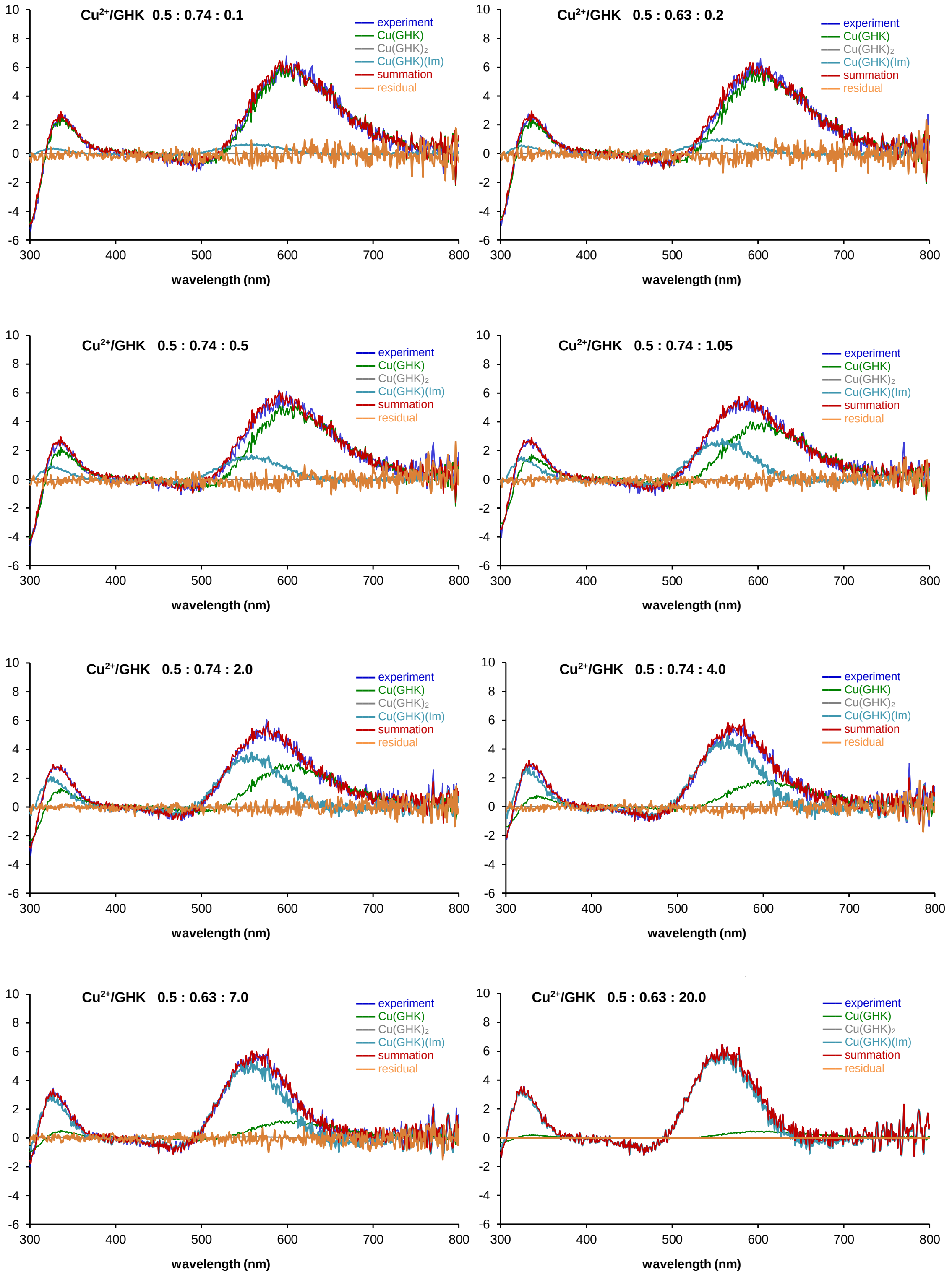

Figure S11. Decomposition of selected CD spectra of Cu/GHK/Im 0.5:0.74:*n* (*n* = 0–20 mM). The corresponding percentages of each species are shown in Figure 3 (main text). *Blue spectrum*, experiment; *green spectrum*, Cu(GHK); *grey spectrum*, Cu(GHK)$_2$; *cyan spectrum*, Cu(GHK)(Im); *red spectrum*, summation of Cu(GHK), Cu(GHK)$_2$, and Cu(GHK)(Im) spectra; *orange spectrum*, difference between the experimental spectrum (blue) and the summation (red).

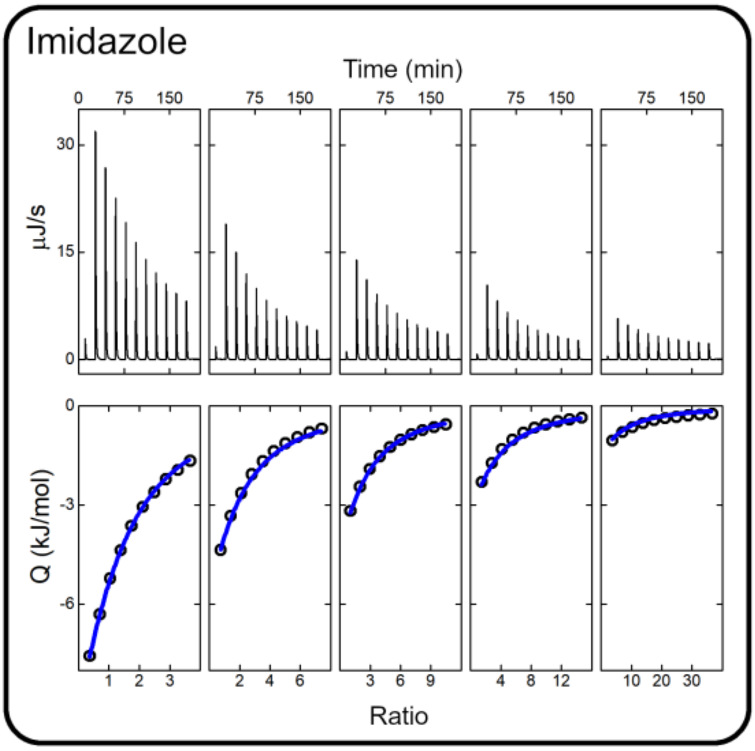

Figure S12. ITC titrations of Imidazole (Im) into solutions containing Cu(GHK): Buffered solutions (20 mM HEPES, 100 mM NaCl, pH 7.4) of 1:0.97 of GHK:$CuCl_2$ was titrated with Im in a range of 0.4 to 40 mol equiv., at 25 °C. The volume of each injection was 24 μL, with concentration of Im in syringe 12 mM. The initial concentrations of GHK in cell were 1.0, 0.5, 0.35, 0.25 and 0.1 mM, respectively, from left. The upper plots show the raw experimental data. The lower plots show heat in each injection (open dots) with fits (blue lines) of ${}^{C}K_{7.4}$ for the ternary Cu(GHK)(Im) complex.

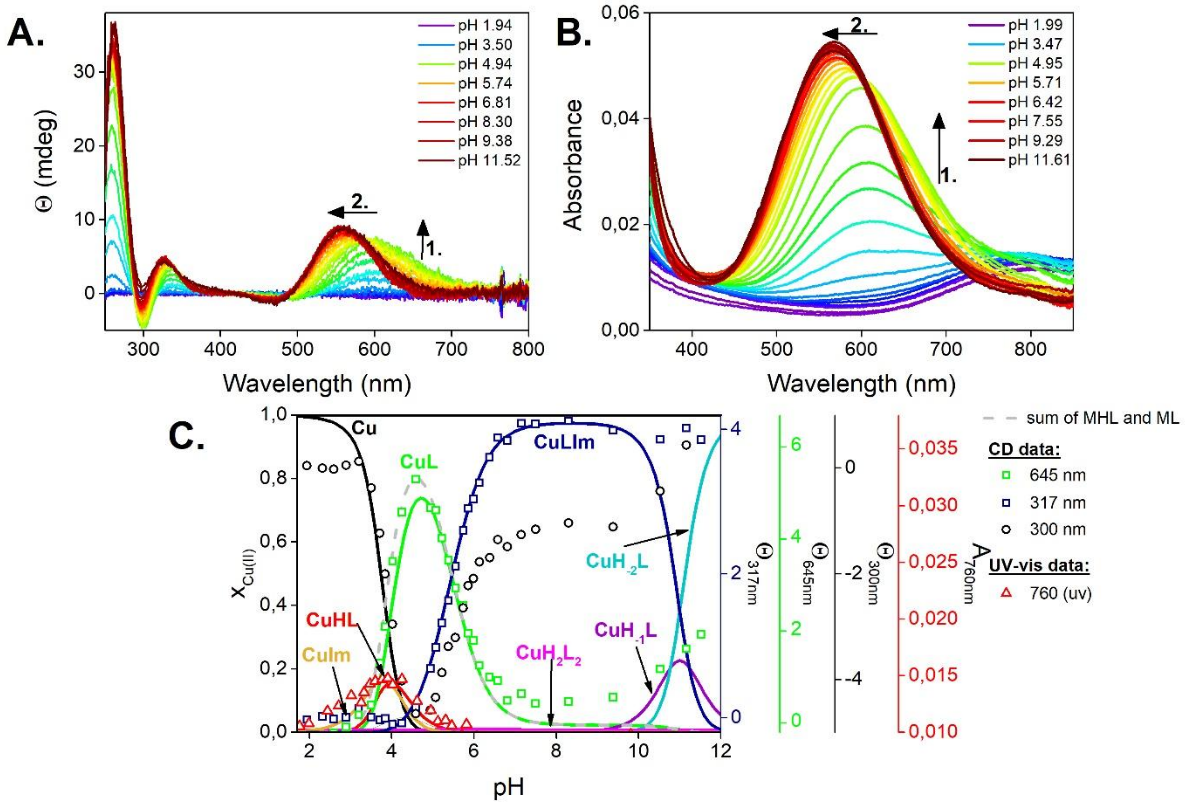

Figure S13. (A) CD and (B) UV-vis spectra of pH-metric titration of 0.95 mM GHK, 0.8 mM $CuCl_2$ and 50 mM Im at room temperature. (C) Species distribution of Cu(II) complexes of Cu/GHK/Im at 25 °C calculated for concentrations used in UV−vis and CD titrations (see above) based on stability constants presented in Table S1. Left-side axis represents molar fractions of Cu(II) complexes, right side axes are absorbance values for UV-vis at 760 nm and ellipticity at 300, 317, and 645 nm.

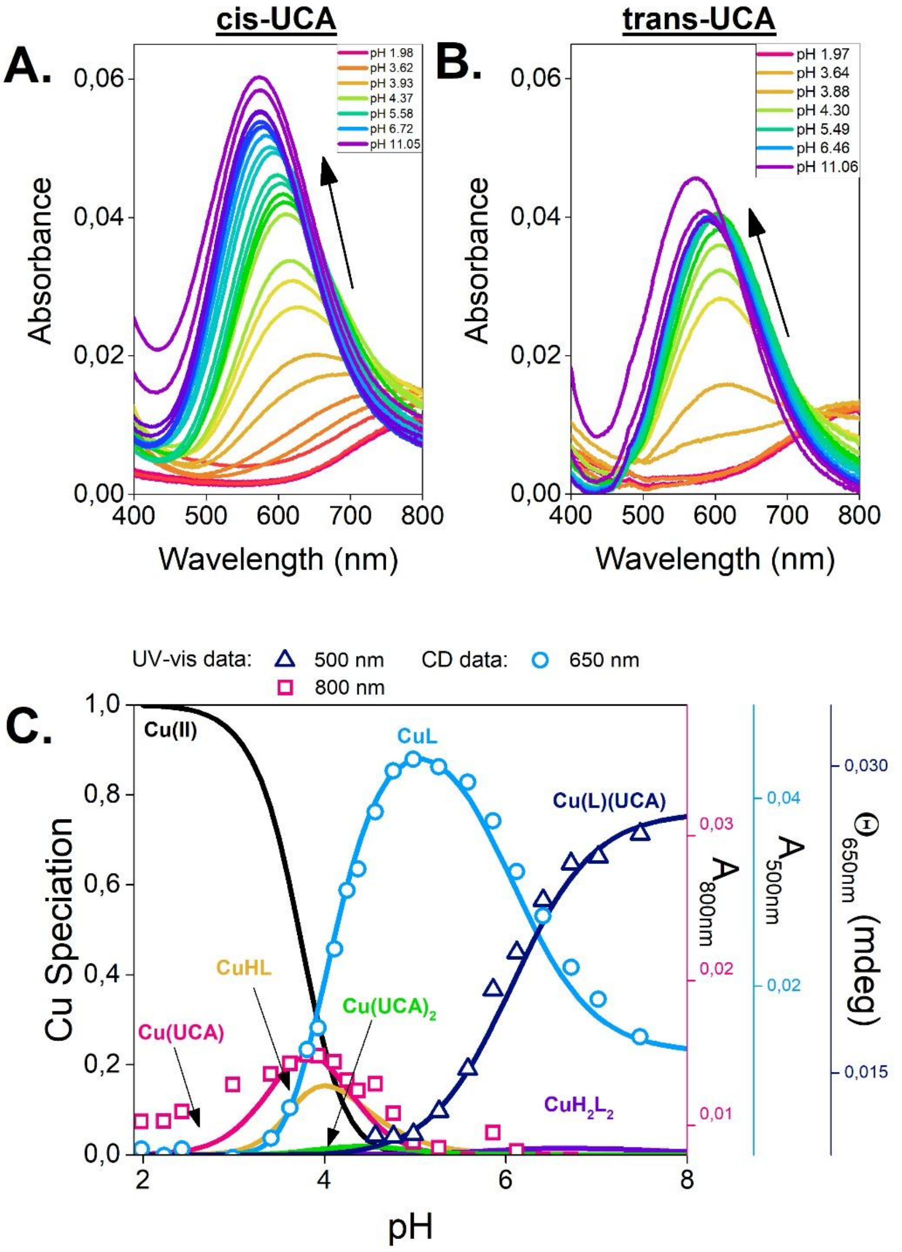

Figure S14. UV-vis spectra of 0.95 mM GHK, 0.8 mM $CuCl_2$ and 6 mM (A) *cis*-UCA and (B) *trans*-UCA. Spectra are color coded from reds (lowest pH) to purples (highest) and additionally direction of spectral changes are marked with arrows. Legend shows selected pH values. (C) Species distribution diagram of Cu(II) complexes of GHK, Cu(II) and *cis*-UCA at 25 °C calculated for concentrations used in UV−vis and CD titrations (0.95 mM peptides and 0.8 mM Cu(II) and 6 mM *cis*-UCA) based on stability constants presented in Table 1 and S1. Left-side axis represents molar fractions of Cu(II) complexes, right side axes are absorbance values for UV-vis at 800 and 500 nm and ellipticity at 650 nm.

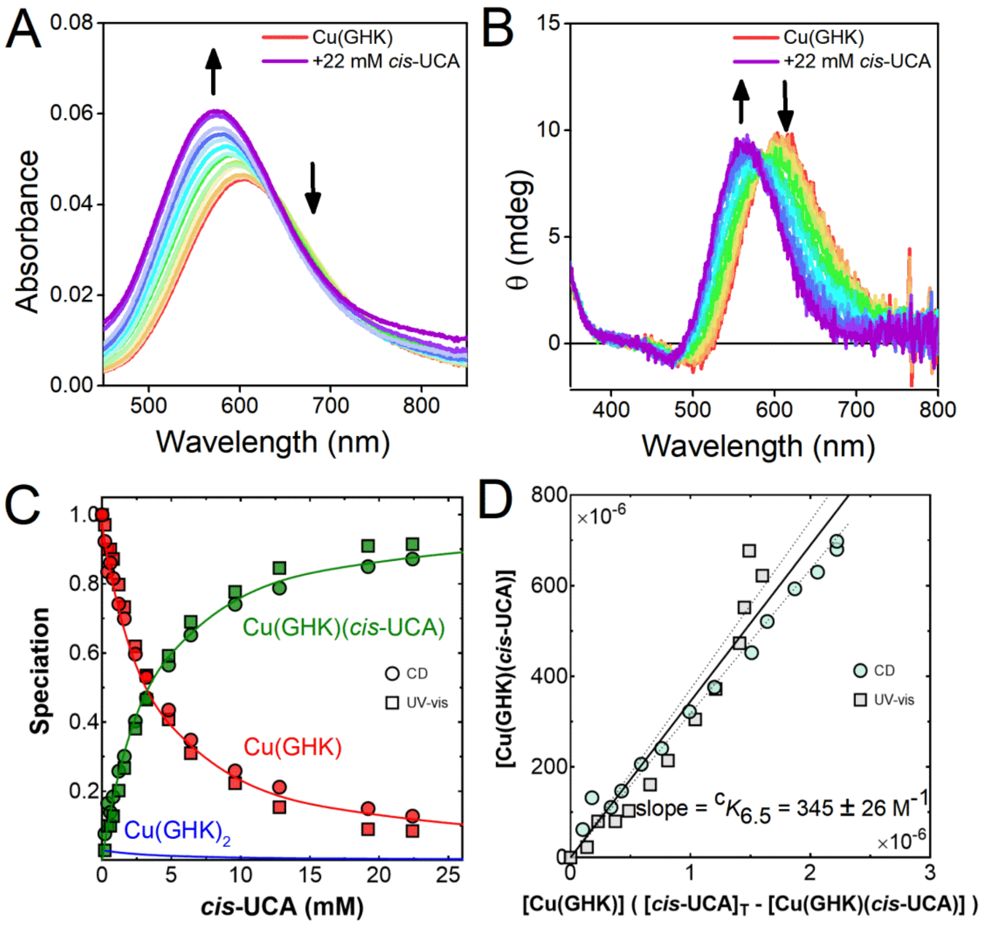

Figure S15. Determination of the conditional Cu(II) binding constant ${}^{c}K_{6.5}$ = [Cu(GHK)(*cis*-UCA)] / ([Cu(GHK)][*cis*-UCA]). (A) UV-vis, and (B) CD spectra of Cu/GHK/*cis*-UCA 0.8:0.95:*n* ($0 \leq n \leq$ 22 mM) at pH 6.5, 25 °C. (C) Speciation of GHK complexes obtained from decomposition of UV-vis and CD spectra in dependence of *cis*-UCA concentration. (D) Determination of the binding constant ${}^{c}K_{6.5}$ using least squares regression. The margin of error derives from the 95% confidence interval (- - - - -).

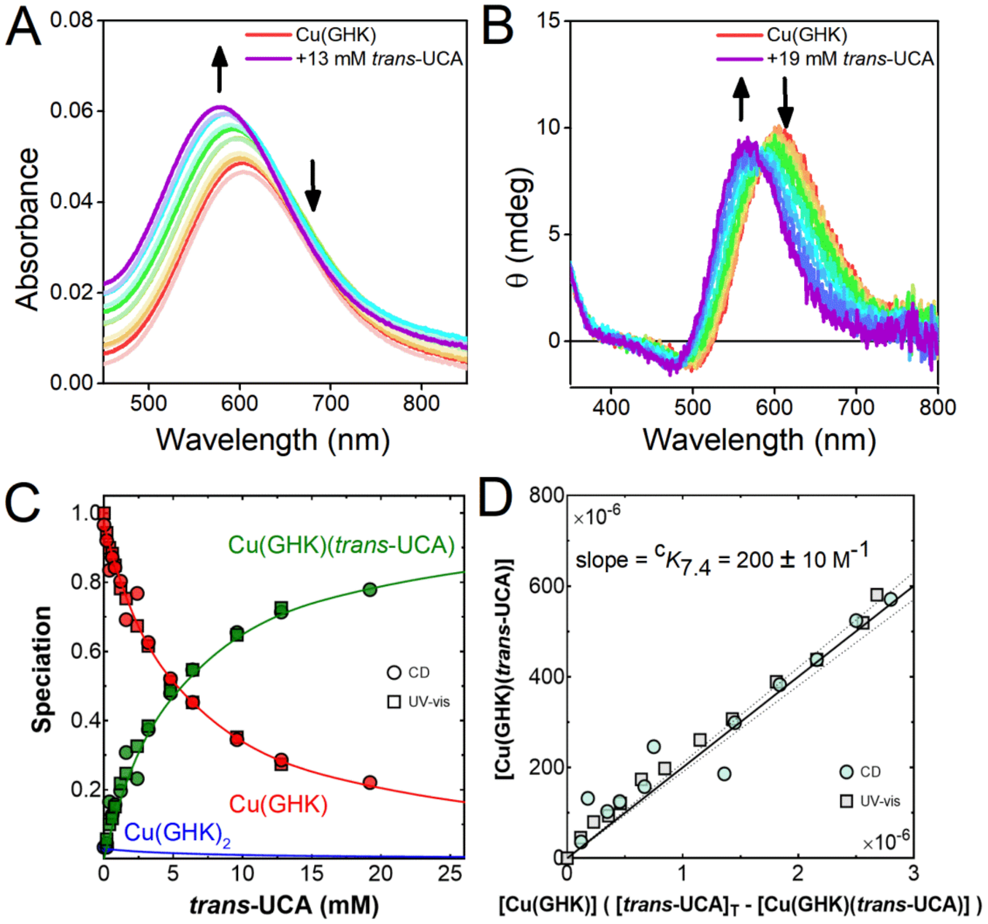

Figure S16. Determination of the conditional Cu(II) binding constant $^{c}K_{7.4}$ = [Cu(GHK)(*trans*-UCA)] / ([Cu(GHK)][*trans*-UCA]). (A) UV-vis, and (B) CD spectra of Cu/GHK/*trans*-UCA 0.8:0.95:*n* (0 ≤ n ≤ 19 mM) at pH 7.4, 25 °C. (C) Speciation of GHK complexes obtained from decomposition of UV-vis and CD spectra in dependence of *trans*-UCA concentration. (D) Determination of the binding constant $^{c}K_{7.4}$ using least squares regression. The margin of error derives from the 95% confidence interval (- - - - -).

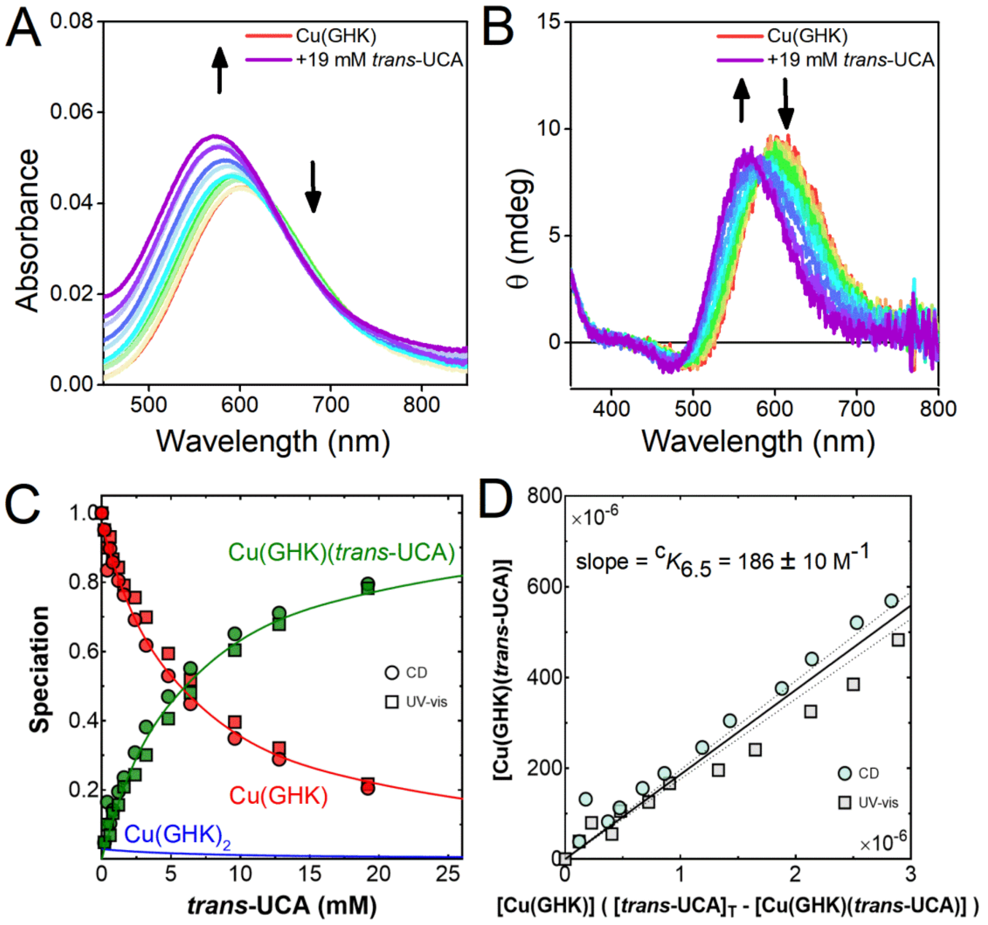

Figure S17. Determination of the conditional Cu(II) binding constant $^{c}K_{6.5}$ = [Cu(GHK)(*trans*-UCA)] / ([Cu(GHK)][*trans*-UCA]). (A) UV-vis, and (B) CD spectra of Cu/GHK/*trans*-UCA 0.8:0.95:*n* (0 ≤ n ≤ 19 mM) at pH 6.5, 25 °C. (C) Speciation of GHK complexes obtained from decomposition of UV-vis and CD spectra in dependence of *trans*-UCA concentration. (D) Determination of the binding constant $^{c}K_{6.5}$ using least squares regression. The margin of error derives from the 95% confidence interval (- - - - -).

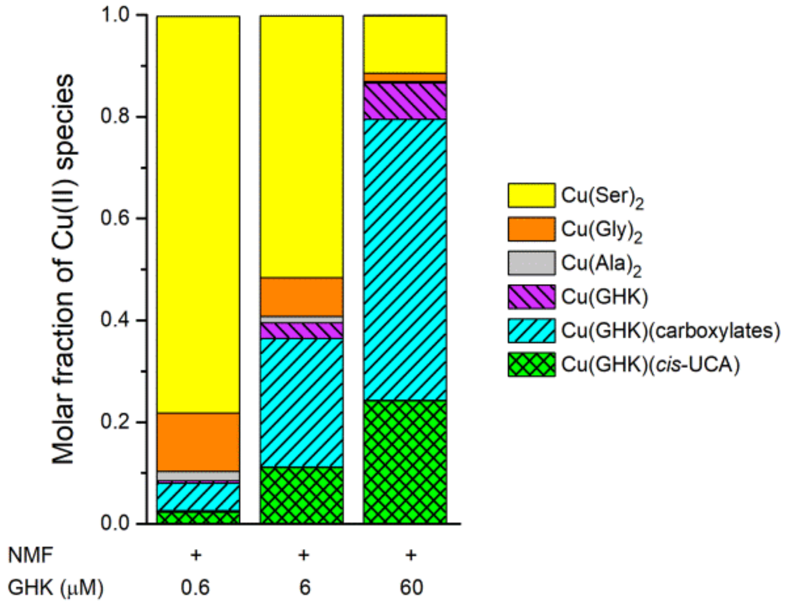

Figure S18. Cu(II) species distribution simulated for NMF, 0.9 μM Cu^{2+} ions, and different concentrations of GHK, at pH 6.5 (referring to the healthy skin). The lowest concentration of GHK (0.6 μM) is equal to the concentrations found in human plasma. Higher concentrations (6 and 60 μM) of GHK may occur after application of cosmetics or other products with GHK as the active substance. The protonation constants and stability constants for Cu(II) complexes were taken from the literature [47,48] and this paper. The conditional constant for the ternary complexes formation of Cu(GHK) with carboxylates (68 M^{-1}) [20] was also included in calculations. Concentrations taken for calculations are 43 mM serine, 30 mM glycine, 23 mM pyroglutamic acid, 18 mM alanine, 14 mM lactic acid, 14 mM *cis*-UCA [11,12]. All ternary complexes of Cu(GHK) with aforementioned compounds with carboxylic groups were combined in the "Cu(GHK)(carboxylates)". Only the species exceeding 1% of all Cu(II) species are shown for clarity.

2. Tables

Table S1. Protonation constants (*K*) for GHK (L) and complex formation constants (β) characterising the interaction of Cu(II) ions with GHK and external ligands, together with the ascribed protonation events

Species	Logβ [1]	p*K*	Protonation event
H_4L	27.652(8)	2.68	$COOH^{C\text{-}term}$
H_3L	24.976(6)	6.43	N^{Im}
H_2L	18.550(5)	7.95	$NH_2^{N\text{-}term}$
HL	10.604(4)	10.60	$N^{\varepsilon\ (Lys)}$
CuHL	20.09(2)		**3N:** $NH_3^{+\ (N\text{-}term)}$, N^{amide}, N^{Im}
CuL	16.50(5)	3.59	**3N:** $NH_3^{+\ (N\text{-}term)}$, N^{amide}, N^{Im}, $COOH^{C\text{-}term}$
$CuH_{-1}L$	6.90(1)	9.61	**3N:** $NH_3^{+\ (N\text{-}term)}$, N^{amide}, N^{Im}, $COOH^{C\text{-}term}$,H_20
$CuH_{-2}L$	-3.87(1)	10.77	**3N:** $NH_3^{+\ (N\text{-}term)}$, N^{amide}, N^{Im}, $COOH^{C\text{-}term}$, H_20, $N^{\varepsilon\ (Lys)}$
CuH_2L_2	37.69(6)		**3N+1N:** $NH_3^{+\ (N\text{-}term)}$, N^{amide}, N^{Im}, $COOH^{C\text{-}term}$ + $N^{Im}(GHK_{ext})$
CuL(Im)	19.43(1)		**3N+1N:** $NH^{3+(Nterm)}$, N^{amide}, N^{Im}, $COOH^{Cterm}$, N_{Im}^{ext}
CuL(*cis*-UCA)	19.29(2)		**3N+1N:** $NH^{3+(Nterm)}$, N^{amide}, N^{Im}, $COOH^{Cterm}$, N_{Im}^{ext}

[1] Values determined by potentiometry at 25 °C and I = 0.1 M (KNO_3). Standard deviations on the least significant digits, provided by HYPERQUAD [36] are given in parentheses.

Table S2. Spectroscopic parameters of binary Cu(II) complexes of GHK, and ternary Cu(II) complexes of GHK and imidazole

	UV-vis		CD		EPR								
Complex	λ_{max} (nm)	ε_{max} (M^{-1} cm^{-1})	λ_{ext} (nm)	$\Delta\varepsilon_{ext}$ (M^{-1} cm^{-1})	$g_\perp$	$g_\parallel$	$A_\perp$ (^{65}Cu)	$A_\parallel$(^{65}Cu)	a^{N1}	a^{N2}	a^{N3}	a^{N4}	τ_c (ns) [1]
							(10^{-4} cm^{-1})						
**Cu(GHK)**	608 [2]	48	608 [2]<br>503 [2]<br>340 [3]<br>300 [4]	+0.44<br>–0.03<br>+0.19<br>–0.22	2.056	2.229	15.3	198.3	10.7	12.2	14.0	n.a.	0.14
**Cu(GHK)$_2$**	568 [2]	68	563 [2]<br>468 [2]<br>328 [3]	+0.50<br>–0.02<br>+0.24	2.057	2.192	22.7	206.2	11.2	13.6	14.0	14.3	0.32
**Cu(GHK)(Im)**	570 [2]	57	557 [2]<br>468 [2]<br>327 [3]<br>298 [4]	+0.30<br>–0.05<br>+0.20<br>–0.06	2.048	2.211	22.7	206.2	11.2	13.6	14.0	14.3	0.13

[1] Isotropic rotational correlation time; [2] *d–d* band; [3] N^{im}–Cu(II) charge transfer band; [4] N^{-}–Cu(II) charge transfer band; ligand hyperfine couplings (a^k) are isotropic; n.a. not applicable

Table S3. Thermodynamic parameters of auto-ternary $Cu(GHK)_2$ and ternary Cu(GHK)(Im) complexes formation determined at pH 7.4 by ITC [1]

	**GHK**	**Im**
$\Delta H_{Cu(GHK)(L)}$ (kJ×mol^{-1})	–19 ± 5 [2]	–25 ± 2
$-T\Delta S_{Cu(GHK)(L)}$ (kJ×mol^{-1})	5	9
$\Delta G_{Cu(GHK)(L)}$ (kJ×mol^{-1})	–14	–16

[1] The results were calculated for the equilibrium $Cu(GHK) + L \rightleftarrows Cu(GHK)(L)$, where L stands for GHK or imidazole.

[2] Errors of the parameter determination are represented by standard deviations.

Table S4. Spectroscopic parameters of binary Cu(II) complexes of *cis*-UCA, and ternary Cu(II) complexes of GHK with *cis*- and *trans*-UCA

	UV-vis		CD	
Complex	λ_{max} (nm)	ε_{max} (M^{-1} cm^{-1})	λ_{ext} (nm)	$\Delta\varepsilon_{ext}$ (M^{-1} cm^{-1})
**Cu(*cis*-UCA)**	709 [1]	39	n.a	n.a.
**Cu(*cis*-UCA)$_2$**	661 [1]	62	n.a	n.a.
**Cu(GHK)(*cis*-UCA)**	571 [1]	73	559 [1]<br>471 [1]	+0.38<br>–0.05
**Cu(GHK)(*trans*-UCA)**	567 [1]	84	559 [1]<br>472 [1]	+0.35<br>–0.04

[1] *d*–*d* band; n.a. not applicable
